# Supplementary material for: Identifying repeat domains in large genomes
Source: Genome Biol. 2006 Jan 31;7(1):R7. doi: 10.1186/gb-2006-7-1-r7 (PMC1431705; doi:10.1186/gb-2006-7-1-r7)
Supplement: Additional File 1 — A zipped file of browsable HTML files with a complete list of the connected components in the repeat domain graph of human Repbase. [file gb-2006-7-1-r7-S1.gz › html/subgraphs/7268.pdf]

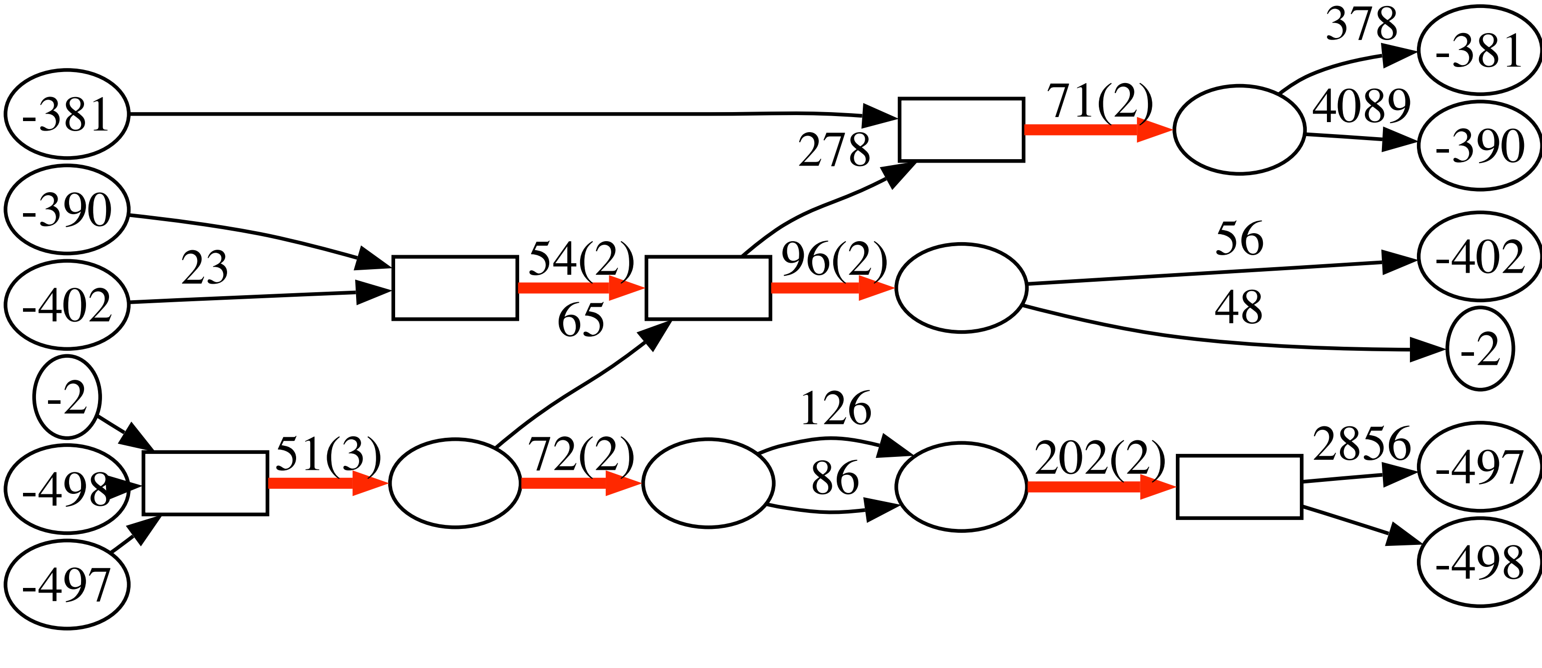

378
-381
71(2)
4089
-381
-390
278
-390
54(2)
96(2)
56
-402
23
-402
65
48
-2
-2
126
51(3)
72(2)
202(2)
2856
-497
-498
86
-497
-498
